# Women’s Family Care Responsibilities, Employment and Health: A Tale of Two Countries

**DOI:** 10.1007/s10834-020-09742-4

**Published:** 2020-12-11

**Authors:** Chiara Mussida, Raffaella Patimo

**Affiliations:** 1 Department of Economic and Social Sciences, Università Cattolica del Sacro Cuore, via Emilia Parmense, 84, 29122 Piacenza, Italy; 2 Department of Economics and Finance, Università degli studi di Bari “A. Moro”, Largo A. S. Scolastica, 53, 70124 Bari, Italy

**Keywords:** Employment, Gender, Family care, Health

## Abstract

Persistently low employment of women in some countries can still be ascribed to a traditional perception of women’s role in society. According to observed data and prevailing social and cultural norms, women have been bearing the primary burdens of housework, childcare, and other family responsibilities. The unequal share of care responsibilities between women and men further worsens the disadvantages of women in balancing public and private life, with an impact on their employment and health outcomes. In this paper we investigate the role of family responsibilities in shaping employment and health outcomes by gender, in Italy and France, during and after the economic downturn. We use data from the European Union Statistics on Income and Living Conditions for the time windows of 2007–2010 and 2011–2014. Our results support that gender differences in the share of responsibilities roles in the public and private sphere influence the employability and health perception of women.

## Introduction

The debate on the relationship between health and labor market outcomes dates back to the seminal work of Grossman (1972), based on Becker’s (1964) analogy between investment in health capital and investment in other forms of human capital to explain differences in individual labor performance. Over the years, the question has remained important because health is thought to be a major determinant of labor force participation, wages, and time use for diverse groups, including men, women, single parents, and older people (for a thorough review, see Currie and Madrian 1999). Following this line of research, we investigate the role that family care responsibilities play in shaping both employment and health outcomes. Specifically, we compared these relationships among adult men and women living in France and Italy, and considered if the relationships changed as a result of the economic downturn of 2007–2008. We concentrate our analysis on data from two sub-periods during and after the 2007–2008 economic downturn to highlight how a further deterioration of employment and health situations of women and men is due to simultaneous economic restrictions and increased loads in family care. In the current context of Covid-19 pandemic, we cannot neglect that possible and further worsening of such conditions might be expected especially for women because they are over-represented in low-paid jobs and care roles (see, for instance, International Labour Organization [ILO] Organization for Economic Co-operation and Development [OECD] and United Nations [UN] 2020).

Worldwide gender participation inequities exist in many fields (e.g., ILO and World Economic Forum [WEF] 2017; European Commission [EC] 2018). Underrepresentation of women is one of the most pressing issues in labor markets and the economic systems of modern societies (Goldin 2006). Gender underrepresentation in social, economic, and political life is most often explained by the overload of family tasks shouldered by women in their private lives (Suh 2016). The combination of underrepresentation in various spheres of public life and overload in private life negatively impacts both employment and income of women (Crompton et al. 2005; Del Boca and Vuri 2007; Hegewisch and Gornick 2011; Wunder and Heineck 2013). Furthermore, social scientists, epidemiologists, and health researchers have noted an additional burden of physical, mental, and psychological stress among caregivers, most often women (European Commission Report 2018). This is especially the case when care is borne by a single family member and involves multiple types of dependents, such as children, persons with disabilities, or older people (Cannuscio et al. 2004; Coe and Van Houtven 2009; Bauer and Sousa-Poza 2015; Dukhovnov and Zagheni 2015).

Beyond the individual impact, there is an economic impact to the gender gap^1^ in the labor market (Cuberes and Teignier 2014). The International Labour Organization (ILO 2017) estimates that reducing the employment gender gap by 25% by 2025, *ceteris paribus*, would increase global employment by 5.3% and thus increase income of women worldwide. When looking at the total work burden (unpaid and paid), women spend more time in work than men, which has an impact on their health status (Dinh et al. 2017). Employment and the health conditions of women are therefore an important consideration in reducing the gender gap in the labor market, especially when taking into consideration the share of family care responsibilities held by each member of a family.

This extensive study on the links between different dimensions related to health and employment of women in Italy and France provides both current and future insights. The investigation of the comparative increase in and persistence of the gender gap adds to our understanding of what factors contribute to or constrain improved gender equity. We have chosen to focus on the situation in Italy and France, given recent changes in welfare and family policies and the relationship to labor market outcomes for women, as reported in the international statistical database and international organization reports. There are also similarities and differences between the two countries that make for an interesting and comprehensive comparison. Similarities include the geographic, demographic and economic characteristics of the two countries, and their close proximity. Moreover, past indicators of the decision-making dynamics of fertility and family formation are also similar (Anxo et al. 2011). However, recent dynamics have diverged considerably between the two countries. For instance, Italy recorded an overall employment rate of around 58% in 2017, while France had an employment rate of 67%.^2^ Of particular interest to the present study, the employment gender gap in Italy was more than twice that of France: 18.2 percentage points (pp) compared to 7.2 pp. (for comparison, the EU28 gender gap was 10.5 pp.). In general, the employment of Italian women ranks low internationally, despite recent socioeconomic changes (Del Boca et al. 2005), including more education for women which is often positively associated with employment (Di Tommaso 1999; Del Boca et al. 2005; Bratti and Staffolani 2012).

An additional difference between the two countries is in time use. According to OECD time use survey data (2017),^3^ although women work more hours per day than men, Italian women are at a particular disadvantage. Italian women not only spend more time working than Italian men, but more time than their French counterparts: 2.2 h in paid work and 5.1 h in unpaid work, compared to 2.9 paid work hours and 3.7 unpaid work hours for French women. Furthermore, the greater involvement of French men in unpaid work reduced the gender gap in France to a third of that in Italy (0.5 h and 1.4 h, respectively). Finally, the funds allocated to family support policies, especially regarding the provision of childcare services, highlights the differences in social preferences between the two countries and subsequent outcomes. While the French “third child policy” boosted fertility rates among French women and family support policy measures adopted therein contributed to an increase in labor market participation among French women, no such effect occurred in Italy due to different policy choices (Del Boca and Wetzels 2008). Regarding childcare expenditures in 2013 alone, France spent three times as much as Italy on early childhood and education care in terms of GDP Italy: 1.3% versus 0.5%.^4^

When we consider both social policy preferences and fertility rates, the differences are even more evident (in 2017, a fertility rate of 1.9 children per woman in France and a fertility rate of 1.3 children per woman in Italy, OECD n.d). Regarding gender allocation of parental leaves, the differences between France and Italy are quite clear. In 2019, both countries had 25 weeks available for maternity and paternity leave in full-rate equivalent weeks (that is, the length of paid leave in weeks at 100% of previous earnings). However, in France this consisted of 19 full-rate equivalent weeks for maternity leave and 5 for paternity leave, whereas in Italy, 25 weeks were for women and five days were for men (Chzhen et al. 2019; UNICEF n.d.; OECD database n.d.). In the time period for our analyses, however, the differences were even more striking: Italy had 25 weeks maternity leave and no paternity leave until 2013 (when it implemented two days), while France already had two weeks paternity leave in 2002, increasing to 28 days (equivalent of 5 full-rate weeks) in 2014.

Regarding childcare, the 2010 EU target of 33% childcare coverage (children between 0 and 3 years of age), reaffirmed by the EU 2020 Strategy, has been attained in both countries. France increased coverage from 44% (2011) to 49% (2016) and Italy from 25% to 35% during the same period, although both countries suffered a temporary reduction during the economic downturn. As recently as 2017, despite increased coverage, a difference in the availability of childcare services existed, which may be attributed to the different attitudes of the two countries. A lack of childcare facilities, in fact, determines to a great extent whether women with children continue to work after maternity leave ends (Romito et al. 2002; Gornick and Meyers 2003; De Henau et al. 2008).

This study adds to the existing literature on the impact of differences in the division of family care responsibilities between women and men on employment and health status by analysing microdata of two countries with similar characteristics but with different paths towards closing the gender gap. Moreover, this work relies on a multidimensional approach in the comparison of gender gaps, which is not commonly found in the study of gender differences. This is relevant because of the overlap between professional and family life. Examining both employment and health allows for further insights into the impact of division of family care responsibilities.

The paper proceeds as follows. In the next section we describe the relevant literature on employment and health status and the links to family care responsibilities. Next, we discuss in sequence: the rationale for our empirical strategy to simultaneously estimate employment and health, the microdata used, and the main estimation results. Finally, we conclude with final observations and remarks.

## Literature Review

For the present study, we focused on family care responsibilities among heterosexual couples. As such, the distribution between women and men within a family is uneven, with considerable differences between paid and unpaid workers in particular. A 2015 survey of EU countries (European Foundation for the Improvement of Living and Working Conditions [Eurofound], 2016) showed that women in families with the youngest child under 7 spent on average, 32 h a week on paid work and 39 h on unpaid work, whereas men spent 41 h a week on paid work and 19 h on unpaid work. Furthermore, when multiple family dependents were present in the same household, such as children and grandparents, both with or without disability, the burden was even heavier. Indeed, children and grandparents with disabilities represent (life)long or medium–short-term family care responsibilities in our modern ageing society, beyond the expected period of time for young children to grow up.

## Family Care Responsibilities and Social Expectations

Since the seminal work of Mincer (1962), several important studies have analysed the impact of time-consuming family care responsibilities on employment and the labor market participation of women. Women’s participation and employment are strongly and negatively affected by the presence of children (Coe and Van Houtven 2009; Dukhovnov and Zagheni 2015; Bauer and Sousa-Poza 2015) as well as marriage itself (Del Boca et al. 2008a, b). Additionally, some studies have investigated the relationship between the double burden of caregiving and employment for mothers with children who have health and/or disability problems and the presence of formal and/or informal childcare (Brandon 2000; Zan and Scharff 2018), finding that the presence of children had a negative effect on the labor market prospects of women.

Some studies have focused on the relationship between the availability of childcare services and the labor market performance of women. Both field-specific and institutional literature looking at the role of family policies have shown that the provision of formal childcare and lower childcare prices are positively associated with the labor market performance of women (Herbst and Barnow 2008). Addabbo et al. (2012), for example, found a positive association between the availability of childcare services and women’s labor market opportunities. The researchers examined European Union Statistics on Income and Living Conditions (EU-SILC) data for Italy in 2007 and noted that, consistent with the literature on female labor supply, the availability of formal childcare services positively affected women's participation as well as their hours of paid work. Informal childcare, that is the unpaid care usually provided by a grandparent of the child or by other relatives, friends or neighbours, however, has been associated with a lower employment propensity of women. Erhel and Guergoat-Larivière (2013) examined twenty-four European countries using the 2005/2006 EU-SILC data and found that women’s employment was positively associated with formal childcare and with characteristics of national labor market regimes, whereas the use of informal childcare was associated with lower employment rates for women.

The presence of older family members in the household has also been studied in terms of the negative effect on women’s employment (Johnson and Lo Sasso 2006; Bolin et al. 2008; Van Houtven et al. 2013), and opportunity cost between intra-family money transfer and employment outcomes (Cox 2003, 2007). It is worth noting however that multiple studies have investigated the impact of childcare provided by grandparents on mothers’ labor market perspectives, and therefore, investigated the joint effect of the presence of children and older family members without disability (grandparents) in the same household (Lewis et al. 2008; Settles et al. 2009). The findings suggest, as an exception to the earlier point about informal care being less effective, that many grandparents provide care for their grandchildren when parents are unable to do so or cannot afford formal paid care, because most of this care is unpaid (Carmichael and Charles 2003; Viitanen 2010; Arpino et al. 2014). The help of grandparents can be crucial for working mothers (and fathers), especially during years when both work and the care of children is very demanding (Tobío et al. 2010). Family care responsibilities also include the possible presence of household members with disabilities. The sparse literature on this topic has mainly focused on wives’ responses to deterioration in their husbands’ health (Berger and Fleisher 1984; Haurin 1989; Charles 1999; Siegel 2006; Parodi and Sciulli 2008; Braakmann 2014) and has found mixed results regarding the existence of a ‘disability employment penalty’ (Berthoud 2008), that is, the impact of living with a person with disabilities on the employment probabilities of that same person’s relatives.

An important determinant of women’s employment then is the distribution of family tasks and responsibilities in the household. This would normally imply that women often have more restricted access to the labor market, with considerable negative consequences to their economic status through a reduction in human capital accumulation and productivity (Klasen 2002; Klasen and Lamanna 2009). Although the dual-earner model (both partners working full time) or the modified breadwinner model (one partner working part-time—the so-called secondary earner—and the other one full-time) have replaced the traditional male breadwinner model in most countries, the gender gap in terms of activity and part-time work remains (Ciccia and Bleijenbergh 2014).

A related gender-biased determinant of women’s employment refers to different expectations of women and men rooted in social institutions and social preferences. Social institutions establish the norms regarding how men and women interact and the choices they make and in so doing differentiate their behaviours (consciously and unconsciously). The public provision of family services is then influenced by these previously established social institutions. A lack of care services and measures has been widely recognized as one of the more persistent obstacles to the equalization of shared family care responsibilities (Del Boca and Vuri 2007; Hegewish and Gornick 2011; Brilli et al. 2016).

## Working Hours and Health

Health is also known to be an important determinant of economic performance and, in particular, employment (Grossman 1972; Currie and Madrian 1999). Family responsibilities not only limit employment opportunities but may also alter the health status of women, further reinforcing the negative effect on both economic factors and employment (Garcìa-Gomez et al. 2010; Stewart 2013). The greater number of hours of unpaid work undertaken by women makes them more susceptible to stress than men, because the unbalanced share of family care responsibilities increases the magnitude of the negative effect of care duties on health and employment: equalizing gender roles and sharing activities would thus improve women’s health (McDonald et al. 2005). Paternity leave, for instance, is correlated with shorter career breaks, longer working hours, fewer penalties in terms of promotions and wages and improved labor market positions for mothers (Pylkkänen and Smith 2004; Keck and Saraceno 2013). Additionally, fathers’ involvement in childcare is positively associated with children’s social, emotional, physical and cognitive development (Tamis-LeMonda and Cabrera 2002; Allen and Daly^2007^).

Studies examining the relationship between employment and health status suggest that health has a pervasive effect on most labor market outcomes, including wages, income, participation, and hours worked (Currie and Madrian 1999). The direct and indirect health effects on women’s employment have also been considered. Both the epidemiological and psychological literature have shown that caregivers may suffer from high stress during an intense period of care, often leading to a worsening of the caregivers’ health (Miller et al. 1991; Hooyman and Gonyea 1995; Gallagher and Mechanic 1996; Pinquart and Sörensen 2011). In particular, the psycho-physical stress faced by women due to multiple burdens has been linked to adverse effects on physical and mental health (Henretta et al. 2002; Do et al. 2014) and is associated with higher economic costs (Pierret 2006; Wiemers and Bianchi 2015; Suh 2016) at both the individual and the collective level.

The majority of studies on health and labor outcomes have focused on the role played by health in retirement decisions (Bound et al. 1999; Au et al. 2005; Disney et al. 2006) and show that a worsening health status accounts for labor market exits. Although relevant studies have investigated the relationships among employment, family responsibilities, and health, individually or in pairs (employment and family responsibilities or health and family responsibilities), few studies analyse these three variables in combination or as direct comparison (i.e., between Italy and France). Some researchers have investigated the relationship between caring activities and labor force participation and found a negative association between them, in both Italy (see for instance, Marenzi and Pagani 2005 and Bratti and Staffolani 2012) and France (see for instance, Kocourková 2002 and Robila 2012). Others have focused on the association between poor health status and marginal/atypical employment (Rodriguez 2002; Bardasi and Francesconi^2004^). The lack of employment opportunities in some southern European countries, such as Italy, has been shown to have negative consequences on female employment and on women’s re-entry into paid work after childbirth in particular (Haas and Rostgard 2011). Similarly, a constrained labor market has been argued to reduce women’s opportunities to return to the labor market (Del Boca et al. 2005). Studies in Italy and France that jointly investigate health and employment effects are limited to maternal health and after-birth labor market re-entry (see Saurel-Cubizolles et al. 2000; Romito et al. 2002).

The novel contribution of this paper is that it provides a comprehensive and direct comparative analysis of three important phenomena in Italy and France. These countries represent interesting cases because of the similarities and differences previously explained, and may be seen as representing different welfare regimes, care regimes, family and employment policies, and social norms (Mussida and Sciulli 2019). Specifically, Italy has a traditional, family-oriented Mediterranean welfare and care regime and combines relatively low expenditures on public caring activities with a predominantly male breadwinner model; thus, informal caregiving is widespread and is usually done by women (Bettio and Plantenga 2004). The existing literature explores the role of a particular kind of informal childcare, that provided by grandparents. Arpino et al. (2014), for instance, found that informal childcare in Italy was positively associated with maternal employment because it substituted for the lack of formal childcare. Italy’s welfare system is traditionally characterized by strong job protection for the head of the household and a low level of transfers amongst the working age population (Kuitto 2011; Fabrizi et al. 2014), as well as a conservative and protectionist role of the family (Bambra and Eikemo 2009; Saraceno 2017), much to the disadvantage of the female population. In contrast, France, like many Western European countries, may be described as having a corporatist welfare state regime and is characterized as providing relatively high financial support for families but more limited support to working parents with young children (Korpi 2000; Leitner 2003; Thévenon 2011). While preserving a conservative profile, the French welfare system is quite effective in providing social policies that help women remain in the labor market through the provision of relatively low-cost public support for caring activities. In France, formal care strategies for both children and the elderly are also well developed. Traditionally, priority is given to services for young children and financial resources, whereas time-off arrangements are relatively underdeveloped (Bettio and Plantenga 2004).

To conclude, the literature review on the effects of family responsibilities (children, elderly persons, persons with disability) separately on employment and health highlight a gap in research on their simultaneous effects. To fill this gap, we conduct a comprehensive and comparative analysis of three important phenomena (i.e., employment, family responsibilities and health) between Italy and France.

## Empirical Strategy, Data and Variables

### Empirical Strategy

We are interested in estimating the impact of health on employment opportunities by gender in Italy and France during and after the economic crisis. Because health status may guide employment decisions, an endogeneity problem due to simultaneity may arise. In order to account for this endogeneity issue, we estimated a two-equation system model using the STATA routine *cmp* (for details, see Roodman 2011). One equation modelled the employment (probability) choice suspected of being endogenous—this was our main equation of interest—while the other modelled the health status and included the employment indicator on its right side. This resulted in a two-equation system model (see Altonji et al. 2005 for a similar application) that can be consistently and efficiently estimated by limited information maximum likelihood. The model allowed us to deal with endogeneity, and we used an indicator for identification purposes (the regional unemployment rates; see the Data Section), which affects the employment decision suspected to be endogenous but not the health status. The simultaneous equation model allowed us to account for endogeneity, and it incorporated instrumental variable (IV) Heckman selection modelling. The advantages of such a modelling framework over a simple IV method or panel data model are the control of the endogeneity issue and (as allowed by the Heckman specification) the consistency and efficiency of the estimates.

We simultaneously estimated two binary (probit) regression models for the probability of being employed and having a good health status by gender in Italy and France, for individuals between 25 and 64 years of age for the period 2007–2010 and 2011–2014, during and after the crisis, respectively. The choice of binary regression models allows obtaining a simplified and convenient representation of employment probabilities for both women and men, as well as the probability of good health. The dependent variable for employment was *one* if the individual was employed and *zero* otherwise. For the health status analysis, the outcome was a binary variable with a value of *one* for good/very good health and *zero* for bad/very bad health or chronic disease (for details on the health status variable, see Variables Section and footnote 5).

The probit model used to estimate the employment equation was derived from a latent continuous variable (${y}_{1}^{*}$) approximated by a discrete distribution and related to a set of explanatory variables $x$ according to a standard linear model that can be represented as follows:

1 $${y}_{1i}^{*}=\beta {x}_{i}+{v}_{i},$$

where $\beta$ is a vector of associated parameters to $x$ and $v$ is an error term drawn from a standardized normal distribution.

While ${y}_{1}^{*}$ is unobserved, ${y}_{1}$ is observed and related to ${y}_{1}^{*}$ by the following relationship:

2 $$y_{{1i}} = \left\{ {\begin{array}{*{20}l} {1if\,y_{{1i}}^{*} } & { > 0} \\ {0.} & {otherwise} \\ \end{array} } \right.$$

The probit model for the health status equation was also derived from a latent continuous variable ${y}_{2}^{*}$ again approximated by a discrete distribution and related to a set of explanatory variables $z$ according to a standard linear model as follows:

3 $$y_{{2i}}^{*} = \alpha y_{{1i}} + \gamma z_{i} + u_{i}$$

where $\alpha$ is the coefficient associated with the endogenous employment variable, $\gamma$ is a vector of parameters associated with $z$, including some $x$ variables, and $u$ is an error term drawn from a standardized normal distribution.

The two-equation system model allows the error terms of both equations to be correlated. Accordingly, we also estimated a correlation term ${\rho }_{vu}$ measuring the correlation between residuals related to health and those of the employment equation. In particular, a positive correlation would indicate that an unobserved term increased both the health and employment outcomes, and vice versa in the case of negative correlation. Finally, for identification purposes we introduced a variable in the employment equation, the regional unemployment rate (for details, see the Section on “Variables”), which explained employment but not health.

### Data

We analysed data from the four successive waves of the EU-SILC survey that took place in the time-periods of 2007–2010 and 2011–2014, during and after the economic downturn respectively, focusing on the Italian and French samples. The survey is conducted in most countries across the European Union by the relevant national institutes of statistics, using harmonized questionnaires and survey methodologies (Eurostat 2010).

Although they follow common guidelines, sampling designs can differ from country to country. In Italy and France, the EU-SILC is a rotating panel survey with 75% overlap of samples in successive years. The non-overlapping sample is drawn according to a stratified two-stage sample design, where municipalities (LAU 2 level, partitions of the regions) are the primary sampling units (PSUs), while households are the secondary sampling units (SSUs). The PSUs are divided into strata according to their population size, and the SSUs are selected by systematic sampling in each PSU. Our analysis considers the longitudinal sample of individuals interviewed in all four successive waves that took place in the two periods mentioned.

Our samples included people between 25 and 64 years of age. In order to avoid confusion over education enrollment and early retirement issues, we excluded from our analysis individuals under the age of 25 years and over the age of 64 years. As a robustness check, we also excluded individuals aged 60 and over from our samples (to completely avoid early retirement decisions), but the results remained basically the same (for the sake of brevity, we do not report the results; they are available upon request), and we therefore retained the conventional 64 years of age as the upper limit of our age range. We dropped individuals with missing values for some variables in our samples by country, time period, and gender. Considering both the non-employed and the employed in the age range examined, for Italy 9373 (7688) female and 9000 (6,893) male observations remain for the period of 2007–2010 (2011–2014) and for France, 12,592 (12,123) female and 11,172 (11,000) male observations remain.

### Variables

Tables 1 and 2 report weighted summary statistics of the variables used in the econometric analysis, computed on the samples of women and men disaggregated by time period for Italy and France, respectively. The dependent variable in our main equation (see above, “Empirical Strategy”) is the probability of being employed. Italian women showed the lowest employment rates with respect to both Italian men and French women. We found that the employment rate for women in Italy was 56.4% (57.9%) in 2007–2010 (2011–2014), compared to 84.1% for men in the first period (78%). Interestingly, French women showed a relatively high employment rate (73.1% in 2007–2010 and 72.3% in 2011–2014) and a lower gender gap compared to Italy. The definitions of employment and non-employment did not match the ILO criteria, however. Indeed, in the EU-SILC questionnaire, the respondents were asked to self-define their main economic status in the current year.^5^ However, the magnitude in employment gender gaps was clear. According to the official statistics (Eurostat n.d.) the employment gender gap in Italy varied between a peak of 25.1 pp. in 2007–2011 and 21.9 pp. in 2011–2014. In France, the gender gap was lower compared to Italy, and it decreased from 10 pp. in the first period to 8.8 pp. after the economic downturn.

**Table 1:** Descriptive statistics of individual and household characteristics for health and employment equations by gender in Italy for the periods of 2007–2010 and 2011–2014

	Women 2007–2010		Men 2007–2010		Women 2011–2014		Men 2011–2014	
	Mean	S.D	Mean	S.D	Mean	S.D	Mean	S.D
Employment equation								
Employed	56.4	49.6	84.1	36.5	57.9	49.4	78.0	41.4
Age [25, 34]	18.3	38.7	18.0	38.4	15.0	35.7	15.0	35.7
Age [35, 44]	31.4	46.4	29.0	45.4	27.8	44.8	16.6	37.2
Age [45, 54]	28.0	44.9	31.7	46.5	32.3	46.7	27.3	44.5
Age [55, 64]	22.3	41.6	21.4	41.0	24.9	43.2	31.6	46.5
Primary education	44.2	49.7	47.3	49.9	34.1	47.4	35.6	47.9
Secondary education	35.7	47.9	35.0	47.7	42.1	49.4	45.2	49.8
Tertiary education	20.1	40.1	17.7	38.2	23.8	42.6	19.2	39.4
Married	71.8	45.0	67.2	47.0	66.0	47.4	64.1	48.0
Presence of children [0, 6]	16.6	37.2	16.4	37.0	14.0	34.7	14.6	35.3
Presence of elderly persons without disability	8.7	28.1	6.9	25.3	6.9	25.4	5.2	22.3
No person with disability in the household	77.4	41.8	77.5	41.8	75.2	43.2	74.8	43.4
Person with disability in the household	15.4	36.1	15.8	36.5	18.2	38.6	18.9	39.1
Person with strong disability in the household	7.2	25.8	6.7	25.0	6.6	24.8	6.4	24.5
Family size^b^	2.0	5.2	1.9	5.5	3.2	7.3	3.2	1.2
Regional unemployment rate	7.44	3.49	7.28	3.44	11.14	4.78	11.1	4.74
2007–2011	25.0	43.3	25.0	43.3	25.0	43.3	25.0	43.3
2008–2012	25.0	43.3	25.0	43.3	25.0	43.3	25.0	43.3
2009–2013	25.0	43.3	25.0	43.3	25.0	43.3	25.0	43.3
2010–2014	25.0	43.3	25.0	43.3	25.0	43.3	25.0	43.3
Health equation^a^								
Good health	84.3	36.4	85.9	34.8	81.0	39.2	83.3	37.3
Employed	56.4	49.6	84.1	36.5	57.9	49.4	78.0	41.4
North	42.1	49.4	43.7	49.6	47.6	49.9	47.6	49.9
Centre	22.0	41.4	22.7	41.9	21.7	41.3	22.6	41.8
South	35.9	48.0	33.6	47.2	30.7	46.1	29.8	45.8
Observations	9373		9000		7688		6893	

*Source* Authors’ calculations on 2007–2010 and 2011–2014 EU-SILC data

^a^For the health equation, we only report the descriptive statistics of the variable not included in the employment equation

^b^Figures are in percentages, apart from family size in units

**Table 2:** Descriptive statistics of individual and household characteristics for health and employment equations by gender in France for the periods of 2007–2010 and 2011–2014

	Women 2007–2010		Men 2007–2010		Women 2011–2014		Men 2011–2014	
	Mean	S.D	Mean	S.D	Mean	S.D	Mean	S.D
Employment equation								
Employed	73.1	44.3	82.5	38.0	72.3	44.7	80.8	39.4
Age [25, 34]	17.1	37.7	16.1	36.7	17.3	37.8	16.9	37.5
Age [35, 44]	28.3	45.0	29.3	45.5	27.1	44.4	28.0	44.9
Age [45, 54]	29.4	45.5	28.8	45.2	29.9	45.8	29.1	45.4
Age [55, 64]	25.2	43.4	25.9	43.8	25.8	43.7	26.0	43.9
Primary education	27.9	44.8	22.1	41.5	21.0	40.7	16.7	37.3
Secondary education	41.1	49.2	50.1	50.0	32.2	46.7	39.0	48.8
Tertiary education	30.9	46.2	27.8	44.8	35.9	48.0	31.6	46.5
Married	62.2	48.5	63.4	48.2	58.2	49.3	57.8	49.4
Presence of children [0, 6]	21.0	50.2	22.3	51.8	21.7	51.5	22.6	52.4
Presence of elderly persons without disability	17.0	37.6	17.8	38.2	17.2	37.7	17.8	38.2
No person with disability in the household	3.9	19.3	1.6	37.8	4.0	19.7	2.0	14.0
Person with disability in the household	11.5	31.9	11.8	32.2	11.4	31.8	12.6	33.2
Person with strong disability in the household	5.9	23.4	5.5	22.7	6.4	24.5	6.3	24.3
Family size^b^	1.9	0.5	1.9	0.6	3.0	1.3	3.0	1.3
Regional unemployment rate	8.35	2.53	8.31	2.47	9.74	0.85	9.74	0.85
2007–2011	25.0	43.3	25.0	43.3	25.0	43.3	25.0	43.3
2008–2012	25.0	43.3	25.0	43.3	25.0	43.3	25.0	43.3
2009–2013	25.0	43.3	25.0	43.3	25.0	43.3	25.0	43.3
2010–2014	25.0	43.3	25.0	43.3	25.0	43.3	25.0	43.3
Health equation^a^								
Good health	68.0	46.7	69.8	45.9	66.4	47.2	68.7	46.4
Employed	73.1	44.3	82.5	38.0	72.3	44.7	80.8	39.4
North	59.2	49.2	58.9	49.2	56.0	49.6	55.2	49.7
Centre	20.9	40.6	23.0	42.1	24.5	43.0	25.6	43.6
South	19.8	39.9	18.0	38.4	19.4	39.5	19.2	39.4
Observations	12,592		11,172		12,123		11,000	

*Source* Authors’ calculations on 2007–2010 and 2011–2014 EU-SILC data

^a^For the health equation, we only report the descriptive statistics of the variable not included in the employment equation

^b^Figures are in percentages, apart from family size in units

The dependent variable for the health equation was the perceived health status (variable PH020 in the EU-SILC code). It was a dummy indicator that equalled *1* for good/very good health and *0* for bad/very bad health or chronic disease.^6^

The overall age range considered [25–64] was divided into four dummy variables for the age brackets [25–34, 35–44, 45–54, 55–64] as these different age ranges were characterized by different employment probabilities as well as different health statuses and different burdens of responsibility.

Given that education plays an important role on employment probabilities as well as on health status (Bratti 2003), we introduced relevant control variables. Educational variables were defined according to UNESCO's International Standard Classification of Education (ISCED). The EU-SILC distinguishes between education completed in the lower secondary stage (ISCED 0–2), upper secondary education (ISCED 3), and post-secondary or tertiary education (ISCED 5–7). In our samples, we found increasing levels of education, especially for women, between the two time periods. This might partly reflect the fact that after the economic crisis, the number of job opportunities increased only in highly skilled professions, and this contributed to modifying the composition of employed workers by educational level both within and between the countries examined (see for instance, van der Ende et al. 2014). There was a reduction in women with primary education (from 44.2% in 2007–2011 to 34.1% in 2011–2014 for Italy, and from 27.9% to 21% for France) and an increase in tertiary educational attainment rates (from 20.1% in 2007–2011 to 23.8% in 2011–2014 for Italy, and from 30.9% to 35.9% for France). We also included an indicator for marital status, which equalled 1 for couples married on a legal basis, for its significant association with employment and health status (Marenzi and Pagani 2005).

The socioeconomic environment may have an impact both on employment probability and on health status (Mussida and Sciulli 2016). Given that in Italy and France, differences in the socioeconomic environment may be appropriately described by geographical discrepancies (García-Gómez et al. 2010; Parodi and Sciulli 2008), the model specification for health included dummy variables for the geographical area of residence (North, Centre, South). More than 40% of the people in the Italian sample lived in the north of Italy, followed by those living in the south (more than 30%) and in the centre (more than 20%). In France, we found that more than 55% of the sample lived in the north, around 25% in the centre, and the remaining 20% in the south. In the employment equation, we included the regional unemployment rate, which, as explained below, was used for identification purposes.

The focus of this work was on the effects of family care responsibilities on women’s employment (and perceived health status). First, family care responsibilities referred to the presence of children in the household. We included an indicator for the presence of children aged between 0 and 6 in our analyses. The data offered the opportunity to distinguish between different age classes of children, and we chose the [0, 6] range because this age class tacitly implies the highest intensity of caring activities (see for instance, Marenzi and Pagani 2005). Second, we included controls for the presence of elderly persons (individuals aged 65 or over) without disability in the household, as they might generate opposite effects: on the one hand, they might need care, but on the other hand they might support the caring activities of other household members, for instance, by taking care of children (see for instance, Arpino et al. 2014). Third, we accounted for possible extra care due to the presence of persons with disabilities with differing degrees of activity limitation in the household. The EU-SILC defines disability as limitation in daily activities of differing degrees (variable PH030 in the EU-SILC code). We used indicators for the presence of household members with both some limitation in activities and strong limitation in activities (Mussida and Sciulli 2019). Similar to what happens with children, differing degrees of disability presumably entail differing degrees of caring duties. Finally, we offered information on family size—i.e., the number of people in a household—as this might affect both the decision to work and the (perceived) health status of women (Baranowska-Rataj and Matysiak 2016). We also examined the impact of such caring activities on the employment probabilities of men to pinpoint differences/gaps and room for improvement.

In the employment equation, we added an indicator to approximate the demand-side effect, namely, the annual regional unemployment rate.^7^ The unemployment rate was used for identification purposes. The estimates of the health equation, as explained above, could be problematic because of the potential endogeneity of the employment decision. To deal with this problem, we estimated a two-equation system model. Our identification strategy relied on the effects of labor market conditions on the employment decisions of women. The regional unemployment rate was thus used for identification because it affected/helped to explain the potentially endogenous variable, that is, the employment probability, but not individual health in Italy and France.

Variations in local labor market conditions have been used as an identification strategy in a number of studies on labor market outcomes, education and training choices and skill acquisition, including, Campolieti et al. (2010), Parent (2006), Riddell and Riddell (2014), among others. The relationship between unemployment and (subjective) health has been extensively studied in the literature, resulting in mixed evidence. Although a positive association between unemployment and distress has generally been found, this relationship differs among population groups and between regions. Young people and the long-term unemployed typically suffer the least from unemployment compared to older people and those who have recently lost their job. Furthermore, the unemployed living in high-unemployment regions are less distressed than the unemployed living in more economically advanced regions (Clark and Oswald 1994). For Italy, as well as for some other European countries (Strandh et al. 2011), the causal effect of regional unemployment on health is due solely to the effect of regional unemployment on individual employment. This was true especially after the economic downturn and the subsequent employment *precarisation* (increase in temporary workers and, in general, disadvantaged workers) and the increase in unemployment (Minelli et al. 2014).

Finally, because we were using panel data, we included yearly dummy variables in our set of covariates. As a limitation of our work, we acknowledge the absence of some information in the longitudinal version of the EU-SILC data that might represent useful additional control variables, such as information on partners as well as on the local availability of childcare services.

## Results

The results for both the employment and health equation are reported as average marginal effects (AMEs). The use of AMEs allowed an interpretation of the effects in percentage terms. Moreover, we carried out specific tests for the equality of AMEs during and after the economic downturn. Those that were statistically different from one period to the other are bolded. All estimates were weighted by using the longitudinal weights provided by the EU-SILC survey (variable RB060 in the EU-SILC code). In the next subsections, we report and comment on the effect of the regressors described above in the Variables section (and in Tables 1 and 2) on employment probability (see Employment Equation section) and health status (see Health Equation) and discuss the changes that we found during and after the crisis.

### Employment Equation

Tables 3 and 4 report the AMEs for the employment equation by gender during and after the economic crisis, for Italy and France, respectively.

**Table 3:** Employment equation for Italian women and men: average marginal effects, 2007–2010; 2011–2014

	Women 2007–2010		Men 2007–2010		Women 2011–2014		Men 2011–2014	
	AME	S.E	AME	S.E	AME	S.E	AME	S.E
Dependent variable: employment probability								
Age dummies: reference–[55, 64]								
[25, 34]	.278	.015***	.229	.011***	**.078**	.018***	**.152**	.016***
[35, 44]	.305	.012***	.262	.009***	**.199**	.015***	**.222**	.016***
[45, 54]	.270	.012***	.251	.008***	**.203**	.014***	**.222**	.011***
Education: reference-primary								
Secondary education	.187	.009***	.046	.008***	**.178**	.011***	**.081**	.010***
Tertiary education	.286	.012***	.082	.010***	**.289**	.014***	**.126**	.014***
Married	− .111	.011***	.052	.009***	− **.068**	.012***	**.096**	.012***
Caring activities: children, household members with a disability, family size								
Presence of children [0, 6]	− .089	.013***	.028	.013*	− .020	.011***	.063	.018***
Presence of elderly persons without disability	− .082	.017***	− .051	.013^†^	− .123	.021***	− .050	.020*
Person with disability in the household	− .027	.013*	− .054	.009***	− **.052**	.013***	− **.060**	.012***
Person with strong disability in the n the in in the household	− .052	.018***	− .045	.012***	.009	.021^†^	− .100	.017***
Family size	− .045	.010***	− .000	.006^†^	− **.032**	.005***	**.009**	.004*
Regional unemployment rate	− .023	.001***	− .004	.001***	− **.018**	.001***	− **.010**	.001***
Yearly dummies								
2008–2012	.022	.013*	.009	.011^†^	.024	.015*	.004	.013^†^
2009–2013	− .046	.013***	− .080	.010***	.052	.015***	.019	.014^†^
2010–2014	− .024	.013*	− .074	.010***	.073	.016***	.034	.014^†^
Observations	9373		9000		7688		6893	
Log likelihood	− 9074.97		− 6418.76		− 7970.72		− 6011.14	

AMEs statistically different from one period to the other are shown in bold

*Note* Average marginal effects, standard errors and significance levels: ^†^p < 0.10, *p < 0.05, **p < 0.01, ***p < 0.001

*Source* Authors’ calculations on 2007*–*2010 and 2011*–*2014 EU-SILC data

**Table 4:** Employment equation for French women and men: average marginal effects, 2007–2011; 2011–2014

	Women 2007–2010		Men 2007–2010		Women 2011–2014		Men 2011–2014	
	AME	S.E	AME	S.E	AME	S.E	AME	S.E
Dependent variable: employment probability								
Age dummies: reference-[55, 64]								
[25, 34]	.274	.013***	.271	.011***	**.231**	.014***	**.200**	.011***
[35, 44]	.299	.011***	.259	.008***	.288	.012***	.236	.010***
[45, 54]	.286	.009***	.246	.007***	.257	.010***	.227	.008***
Education: reference-primary								
Secondary education	.105	.008***	.056	.007***	**.136**	.010***	**.052**	.009***
Tertiary education	.226	.010***	.126	.009***	**.210**	.009***	**.130**	.010***
Married	− .015	.008*	.029	.007***	**.002**	.008^†^	**.029**	.008***
Caring activities: children, household members with a disability, family size								
Presence of children [0, 6]	− .135	.011***	.015	.011^†^	− **.082**	.013***	**.035**	.012***
Presence of elderly persons without disability	− .105	.018***	− .078	.020***	− **.077**	.018***	− **.196**	.020***
Person with disability in the household	− .011	.011^†^	− .036	.009***	− **.016**	.012^†^	− **.041**	.010***
Person with strong disability in the household	.001	.001^†^	− .038	.012***	− **.044**	.015***	− **.074**	.012***
Family size	− .055	.008***	.047	.006***	− **.033**	.004***	**.020**	.003***
Regional unemployment rate	− .007	.001***	− .004	.001***	− **.003**	.001***	− **.009**	.005*
Yearly dummies								
2008–2012	− .011	.010^†^	− .018	.009*	− .009	.012^†^	.003	.010^†^
2009–2013	− .079	.010***	− .086	.009***	− .025	.013*	− .004	.011^†^
2010–2014	− .077	.010***	− .093	.009***	.043	.014***	.025	.013*
Observations	12,592		11,172		12,123		11,000	
Log likelihood	− 13250.69		− 9984.72		− 13542.39		− 10819.31	

AMEs statistically different from one period to the other are shown in bold

*Note* Average marginal effects, standard errors and significance levels: ^†^p < 0.10, *p < 0.05, **p < 0.01, ***p < 0.001

*Source* Authors’ calculations on 2007–2010 and 2011–2014 EU-SILC data

We found an inverse U-shaped relationship between age and the employment probabilities of both genders and the differences between age ranges differed across genders. This points to the importance of analysing and considering dummy variables for each age range (rather than a continuous variable for age). Specifically, we found higher discrepancies between relatively younger women (between 25 and 34 years of age) and relatively older women (between 55 and 64 years of age) compared to (corresponding) differences between younger and older men. Age is therefore a crucial factor when analysing female employment probabilities.

Employment probabilities were positively associated with education in both countries. Interestingly, in Italy we found that female employment was more affected by education than was male employment. The positive role of education for women was supported by similar studies on Italian female labor force participation (Di Tommaso 1999; Del Boca et al. 2005; Bratti and Staffolani 2012). The employment probabilities of Italian women with secondary education were, on average, 18% higher with respect to those with primary education, and the percentages increased to around 29% for tertiary-level-educated ones (+ 28.6% in 2007–2010, during the downturn, with a statistically significant increase to + 28.9% in 2011–2014, after the downturn). The gender gap with respect to the positive role of education was higher in Italy compared to France (see Table 4). There was evidence, especially in Italy, that women who stayed out of employment were those who would have earned the lowest returns from the labor market, with a higher probability than that of men. This is in line with the existing literature showing that female participation rates in Catholic countries such as Italy and Spain, as well as in Greece, are low and concentrated among highly educated women (Blau and Kahn 2003).

Italy and France showed an interesting similarity across gender for the impact of marriage. In both countries, we found that the status ‘married’ had opposite effects on work participation in men and women. Employment probabilities for women were negatively associated with marriage (− 11.1% in 2007–2010 and − 6.8% in 2011–2014 in Italy, and − 1.5% in 2007–2011 in France), while probabilities for men were positively associated with marriage (5.2% in 2007–2010 and 9.6% in 2011–2014 in Italy, and 2.9% in France in both periods).

Moving on to caring responsibilities, the presence of children aged between 0 and 6 years reduced the employment probability of women, especially during the economic downturn and in France, where we noted a significantly different effect of the presence of children during and after the downturn (reducing from − 8.9 to − 2% in Italy from 2007–2010 to 2011–2014 and ranging from − 13.5 to − 8.2% in France during and after the crisis). Male employment probabilities were instead positively associated with the presence of children in both countries. This difference in sign for the impact of the presence of children between genders, and especially the negative effect on the employability of women, is supported by the existing literature (e.g., Addabbo et al. 2012; Erhel and Guergoat-Larivière 2013). Moreover, the negative effect of the presence of children on female employment pinpointed that childcare coverage as well as childcare expenditure, as previously discussed, was not enough to allow women of both countries to fully participate in the labor market.

The presence of older persons without disabilities, where significant, negatively affected the employment probabilities of both genders. To better explain the sign and significance of the presence of older persons and children in the household, we estimated, where possible (sample sizes), the joint effect of the two indicators on employment by using interaction variables.^8^ Interestingly, we found that for women, when either an older person or a child was present, the impact on employment was negative, suggesting that women per se act as care-givers. In contrast, when both were present, the effect on the employment probabilities of women in both countries was not significant, suggesting that older persons in the household likely helped take care of the children. The same negative effect of the older person on employment probabilities was found for men, but a positive impact was found for the presence of children. This might be due to the fact that often the burden of childcare is almost entirely borne by women (Coe and Van Houtven 2009; Dukhovnov and Zagheni 2015; Bauer and Sousa-Poza 2015). Again, when both types of dependents were present, there was no significant effect on the employment probabilities of men in either country. This suggests that quite often, older persons (without disabilities) are a source of (free) informal care that can alleviate the overall responsibilities of both men and women, possibly reducing the total cost of external childcare (see for instance, Carmichael and Charles^2003^; Viitanen 2010; Arpino et al. 2014).

The presence of persons with disabilities in the household (with some or strong activity limitations) negatively affected employment probabilities in both countries. Specifically, the impact was higher for men compared to women, especially in France. This is supported by the existing literature on the indirect employment effects of disability, and specifically, the effects of the presence of a cohabiting individual with a disability on the employment probabilities of the other household members (see, for instance, Berger and Fleisher 1984; Haurin 1989; Charles 1999; Siegel 2006; Parodi and Sciulli 2008; Braakmann 2014).

Family size exerted an opposite effect on the employment probabilities of men and women. The larger the household, the lower were the employment probabilities of women and the higher were the employment probabilities of men. This might reflect the gender roles in the private sphere (division of responsibilities and work within the household) discussed above, as women are primarily responsible for family care while men are primarily responsible for paid labor market activity.

To sum up, the estimates suggest that family care responsibilities negatively and significantly affected employment probabilities, especially for women, and the effect only slightly changed immediately after the economic downturn. Our findings are in line with similar previous work examining the effect of caring activities on labor force participation and employment in Italy and France (for Italy, see, Marenzi and Pagani 2005 and Bratti and Staffolani 2012; for France, see Kocourková 2002 and Robila 2012).

As regards demand-side factors, a high (regional) unemployment rate (used for identification purposes) reduced employment probabilities, and this was in line with expectations. The reduction was even more important after the economic downturn.

### Health Equation

The AMEs of the probit model for the health status of Italian and French women and men are reported in Tables 5 and 6. Interestingly, we noted that employment exerted a mixed role on perceived health status in that employment negatively affected the subjective health status of Italian women (and did not exert a role on the health status of Italian men), while there was a positive association between employment and the health statuses for both women and men in France. The disadvantage of women in Italy was supported both by the official data on employment rate that, as previously explained, pinpointed a worse situation for Italian women with respect to both Italian men and French women, as well as by the time-use survey gathered by the OECD. The effect of employment on health was even more important after the crisis (statistically significant differences between the AMEs for both genders and countries).

**Table 5:** Health equation for Italian women and men: average marginal effects, 2007–2011; 2011–2014

	Women 2007–2010		Men 2007–2010		Women 2011–2014		Men 2011–2014	
	AME	S.E	AME	S.E	AME	S.E	AME	S.E
Dependent variable: health status								
Employed	− .232	.047*	.014	.075^†^	− **.121**	.092*	**.019**	.164^†^
Age dummies: reference-55, 64]								
[25, 34]	.203	.019***	.164	.032***	**.214**	.017***	**.204**	.036***
[35, 44]	.158	.018***	.131	.034***	**.134**	.024***	**.125**	.047***
[45, 54]	.125	.017***	.065	.032*	.074	.025***	**.069**	.046^†^
Education: reference-primary								
Tertiary education	.090	.019***	.024	.013*	**.028**	.014*	**.012**	.026^†^
Married	.000	.011^†^	.027	.011*	**.082**	.035*	**.013**	.021^†^
Geographical area of residence: reference-South								
North	− .003	.013^†^	− .013	.009^†^	− **.042**	.011***	− **.038**	.022*
Centre	.022	.012*	.029	.010***	− .036	.013***	− .004	.020^†^
Caring activities: children, household members with a disability, family size								
Presence of children [0, 6]	− .014	.013^†^	− .016	.012^†^	− .032	.015*	.013	.017^†^
Presence of elderly persons without disability	− .007	.014^†^	− .024	.016^†^	.011	.022*	.017	.024^†^
Person with disability in the household	− .073	.010***	− .057	.011***	− **.117**	.011***	− **.102**	.011***
Person with strong disability in the household	− .121	.014***	− .104	.014***	− .102	.017***	− .052	.028***
Family size	.013	.009^†^	.027	.008***	**.025**	.006***	**.021**	.005***
Yearly dummies								
2008–2012	.003	.011^†^	− .015	.010^†^	.036	.012***	.037	.013***
2009–2013	− .021	.012*	− .018	.013^†^	.035	.012***	.027	.013*
2010–2014	− .029	.012*	− .015	.013^†^	.053	.013***	.030	.013*
Rho	− .628	.094***	− .235	.201^†^	.396	.230***	.170	.412*
Observations	9373		9000		7688		6893	

The AMEs statistically different from one period to the other are shown in bold

*Note* Average marginal effects, standard errors and significance levels: ^†^p < 0.10, *p < 0.05, **p < 0.01, ***p < 0.001

*Source* Authors’ calculations on 2007–2010 and 2011–2014 EU-SILC data

**Table 6:** Health equation for French women and men: average marginal effects, 2007–2010; 2011–2014

	Women 2007–2010		Men 2007–2010		Women 2011–2014		Men 2011–2014	
	AME	S.E	AME	S.E	AME	S.E	AME	S.E
Dependent variable: health status								
Employed	.116	.045**	.113	.042***	**.177**	.024***	**.189**	.036***
Age dummies: reference-[55, 64]								
[25, 34]	.136	.023***	.228	.027***	**.105**	.037***	**.231**	.030***
[35, 44]	.072	.022***	.161	.025***	.047	.040*	.217	.035***
[45, 54]	− .008	.021^†^	.095	.024***	.004	.036^†^	.155	.038***
Education: reference-primary								
Secondary education	.070	.012***	.062	.011***	**.040**	.023***	**.050**	.013***
Tertiary education	.118	.017***	.068	.015***	**.067**	.031***	**.107**	.020***
Married	.044	.009***	.006	.010^†^	**.019**	.009^†^	− **.006**	.011^†^
Geographical area of residence: reference-south								
North	− .002	.010^†^	.004	.011^†^	**.016**	.011^†^	**.013**	.011*
Centre	.013	.012^†^	.013	.013*	− **.012**	.012^†^	**.011**	.012^†^
Caring activities: children, household members with a disability, family size								
Presence of children [0, 6]	.036	.015**	− .020	.013^†^	**.097**	.015***	**.008**	.014^†^
Presence of elderly persons without disability	.041	.022*	.002	.034^†^	**.055**	.023*	− **.064**	.051*
Person with disability in the household	− .113	.012***	− .096	.013***	− **.086**	.014***	− **.092**	.012***
Person with strong disability in the household	− .097	.017***	− .109	.018***	− **.111**	.019***	− **.127**	.017***
Family size	.082	.009***	.043	.009***	**.020**	.005***	**.028**	.004***
Yearly dummies								
2008–2012	− .031	.011***	− .024	.012*	.005	.012^†^	.010	.012^†^
2009–2013	− .031	.012*	− .030	.013*	− .003	.012^†^	.002	.012^†^
2010–2014	− .021	.012*	− .025	.014*	.009	.014^†^	.031	.013^†^
Rho	− .027	.047**	.323	.089**	− .123	.046**	.538	.207*
Observations	12,592		11,172		12,123		11,000	

The AMEs statistically different from one period to the other are shown in bold

*Note* Average marginal effects, standard errors and significance levels: ^†^p < 0.10, *p < 0.05, **p < 0.01, ***p < 0.001

*Source* Authors’ calculations on 2007–2010 and 2011–2014 EU-SILC data

We noted similarities across countries for the individual/household characteristics positively affecting the health status of women and men. Being younger, highly educated (secondary), married and living in a larger household positively affected one’s health status. The relevance and signs were maintained, and most of them were even stronger after the crisis (see AMEs in bold in Tables 5 and 6). In Italy, we found that health status was positively associated with residing in the south, while in France, we did not find a clear role for the macro region of residence.

As far as family care responsibilities were concerned, the presence of children aged from 0 to 6 years exerted a negative impact on women’s health after the economic recession in Italy (-3.2% in 2011–2014), while the impact was positive for French women both during and after the crisis (+ 3.6% during and + 9.7% after the recession, and the difference between the AMEs is statistically significant). There was no association between the presence of children in the household and men’s health. The presence of cohabiting older persons without disability did not affect health in Italy, while it exerted a positive impact on the health of both men and women in France, thus deserving further research into possible explanations. Caring for persons with a disability, whether with some or a strong limitation in activities, negatively affected the health of women and men in both time periods (see Tables 5 and 6).

According to our findings from both the employment equation and the health status analysis, family care responsibilities negatively affected not only the employment probability, but also (and significantly) the perceived health status (especially) of women. Our estimation results also revealed that employment was endogenous in the health equation. The estimated *rho* parameters were significant for both countries and interestingly, mixed between genders. On the one hand, the negative sign of the *rho* parameters for women in France (and in Italy in 2007–2011) suggested that confounding factors, such as individual motivations, preferences and attitudes, increased the employment probability of women and negatively affected health status. For instance, motivations, preferences and good attitudes towards work might increase the labor market participation of women, and this might cause a deterioration in their health status because women must also bear stress and fatigue due to other care responsibilities. On the other hand, the positive sign of the *rho* parameters for men in Italy, and especially in France, suggested that the mentioned confounding factors that increase the employment probability also positively affected health status. These gender differences in attitudes reflect social and cultural norms, which constrain women to bear the primary burdens of housework, childcare and other family responsibilities. These family-care activities, combined with paid work, unavoidably cause stress and deteriorate women’s health. For men, instead, paid work is associated with improved health because they do less unpaid caring activities and thus don't experience the stress of having to do this work and their paid work. It is therefore essential to consider endogeneity, and our results confirm the adequacy and appropriateness of the model strategy adopted.

## Concluding Remarks

In all industrialized countries, the labor force participation rate of women has increased rapidly over the past decades. Nonetheless, it continues to stand well below that of men. Moreover, women in most countries continue to have a discontinuous pattern of employment over their life course, resulting in substantial income loss and more stress from family*–*work conciliation issues.

In order to capture the relationship between employment, family care responsibilities and health outcomes for women (and men, in order to have a clear picture of the gender gap), in this work we simultaneously analysed employment probabilities and health status by gender in Italy and France. We find that employment, especially for women, is negatively associated with family care responsibilities, such as the presence of children aged between 0 and 6 years in the household, as well as elderly persons and persons with some or strong disabilities. The effect on women and men’s employment probabilities only changes slightly after the economic downturn.

Summing up the analyses we ran for the two countries, we note that employment probabilities are positively associated with education in both countries. Interestingly, especially women’s employment in Italy benefits from higher education. We also interpret this as a possible strong channel of reversing the cultural norms and socio-institutional barriers that we found can account for a big difference between two countries’ societies and economies. This interpretation is supported by the finding that the presence of children as well as older persons still has a negative impact on the employment performance of women in both countries, implying that the trade-off is entirely on women’s shoulders, while having the effect of promoting employability for men in Italy and France.

The findings offer strong support for several interventions to help families in the division of family responsibilities, especially if we consider the effect that such intervention exerts on both employment probability and health. Interestingly, we find that the presence of children positively affects health for French women (implying that children are a source of health/happiness/non-stress). In addition, interestingly, the presence of children has no impact on men’s health. The presence of older persons, instead, negatively affects the employment probabilities of both genders. A further family responsibility, that is, the presence of people with disabilities, has a negative impact on both of the studied phenomena, suggesting that extra effort is needed in implementing measures aimed at helping families with members with disabilities in both countries.

The increased availability of specific variables, such as information on partners as well as local availability of childcare services, that we identify as a limitation to our research, could be helpful in developing future research possibilities. These might include also longitudinal studies and, with the availability of the information mentioned, would allow improving the knowledge of the effect of both informal and formal care on employment and health of both women and men.

The need for this line of research becomes even more evident as a result of the recent COVID-19 pandemic, where family responsibilities have fallen even more heavily on women. Recently issued statistical data highlighted the reduction of labor supply and the increase of physical and mental stress (UN 2020). In a further extension of our research, it would be interesting to investigate the effects on employment and health caused by the current COVID-19 pandemic crisis. In fact, while many studies worldwide (Alon et al.^2020^; Berkhout and Richardson 2020; Collins et al. 2020; Power 2020) are concentrating efforts on measuring the different impact of telecommuting work and home schooling on working couples, not so many take a simultaneous look at employment and health outcomes. Nevertheless, studies already reckon that working women bear the brunt of the increased time needed for household chores and childcare, while male counterparts are more likely to be spending more time with the children in more gratifying family work rather than chores (Del Boca et al. 2020). This clearly can carry extra mental and physical stress for women, putting back the clock by several decades, increasing the urgency to assess employment and health outcomes simultaneously, as we have done in this study when Covid-19 pandemic was not at stake.

## Footnotes

1 The gender gap is defined by ILO as the difference in any area between women and men in terms of their levels of participation, access to resources, rights, power and influence, and remuneration and benefits (ILO 2007).

2 Figures available online at http://appsso.eurostat.ec.europa.eu/nui/show.dodataset=lfsq_ergan&lang=en.

3 Figures available at http://stats.oecd.org/index.aspx?queryid=54757#.

4 Data from http://www.oecd.org/els/family/database.htm.

5 The variable PL031 contains information on the self-defined economic status. People are asked whether they are working, unemployed, students, in retirement, have disabilities, are in military service or fulfill domestic tasks.

6 The variable health status is self-perceived. This self-declared definition may lead to self-reporting bias and, therefore, possibly to measurement errors. We explicitly test for this issue by following Contoyannis et al. (2004), and the hypothesis of self-reporting bias (and measurement errors) was rejected. The EU-SILC data also offer a variable for health (PH010) with five categories, ranging from very bad to very good health. Given that we also investigate the issue by gender and time period, we did not maintain a sufficient sample size to allow estimates. We therefore considered a binary representation of health that also allows keeping the interpretation of results simple. In detail, at the upper end of the scale (no chronic disease, PH010 equals 0) we find good/very good health, while bad/very bad are at the lower end (PH010 equals 1). Fair health is an intermediate category based also on cultural interpretation and pertains either to the good/very good or bad/very bad health category (PH010 equals either 0 or 1).

7 Figures available at http://ec.europa.eu/eurostat/tgm/table.do?tab=table&init=1&plugin=1&language=en&pcode=tps00203

8 For the sake of brevity and because for some subsamples (by gender and time period) the estimate was not possible due to small sample size, we do not report the results including the interaction between presence of children and elderly persons. Nonetheless, we discuss the sign and significance for the subsamples allowing such inclusion.
